# Hospital Expenditure. — The Commissariat

**Published:** 1895-11-23

**Authors:** 


### The Institutional Workshop.

### HOSPITAL EXPENDITURE.—THE COMMISSARIAT.

### VII.—THE DOCTORS' TABLE.

With few exceptions all meals served to inmates of an institution are prepared in one kitchen and by one set of servants. In estimating the cost of victualling any part of the household no allowance whatever is made for the rent of premises in which the food is prepared or served, for the wages and board of servants, for the wear and tear of kitchen and table necessaries, for the washing of table linen, &c., or for fuel and lighting. All these items, in addition to a percentage of profit fluctuating according to numbers, amount in hotel and restaurant charges to at least two-thirds of the bill, leaving but one-third for cost of provisions. Anyone who will take the trouble to analyse the exact cost of the food at an ordinary eating-house will at once perceive this to be true. It is evident then that in hospitals where for any reason it has been found necessary to contract for officers' board outside, the cost may reasonably be expected to come to at least twice as much as in hospitals where the doctors' table is supplied from the ordinary kitchen. It is to the cost of the latter arrangement, minus all the above items, that attention is directed.

One or two points stand out from a careful consideration of ascertained facts: (1) That in large hospitals where there are many resident medical officers these are boarded at a far higher rate than in smaller institutions where there are but one or two, and this, although the same gentlemen pass directly from the one class of institution to the other, and cannot be supposed to require better food in one place than in another. (2) That the cost of food for the medical officers in many institutions is out of all proportion greater than that of other members of the household.

The first problem is not difficult of solution. Provincial hospitals employing the services of but one or two house surgeons do not, as a rule, provide an entirely distinct bill of fare for this section of the household. Taking their meals separately, but at the same hours as the rest of the household, they share the same food, and any extras supplied are so insignificant as to raise the average to no perceptible extent. Now the average cost of food per head for the household, including in this term the secretary, steward, and other residents, in several specimen well-dieted hospitals is 8s. per head, there or thereabouts.

Bearing this in mind we are face to face with the second problem, why when a separate table is provided for eight, ten, or twelve doctors the cost of provisions should work out at three or four times the amount for the rest of the household.

We quote two bills of fare side by side as actual specimens of the board provided in two large London hospitals for doctors and nurses respectively. They will be readily recognised as typical. No. 1 works out at from 21s. to 35s. a week; No. 2 at 8s. a week.

| 1. Doctors. | 2. Nurses. |
|---|---|
| *Breakfast.* | |
| Tea or coffee; bread and butter, fish or eggs, bacon and cold meat. | Tea, coffee, or cocoa; porridge, ham, bacon, sausages, fish, or eggs; jam, marmalade, or potted meat. |
| *Luncheon.* | *Dinner.* |
| Cold meat, ham or tongue, or cold pie; cheese, bread and butter, and half-pint of beer. | Joint, vegetables, milk pudding; stewed fruit, jam tart, &c.; bread and cheese; ale or stout, soda water or lemonade. |
| *Dinner.* | *Tea.* |
| Joint, vegetables, sweet, cheese, butter, bread, beer; soup or fish two days a week in addition; poultry once a week. | Tea, bread, butter, jam, plain cake. |

| *Supper.* | |
|---|---|
| Cheese, butter, roll. | Cold meat with pickles or salad; or occasionally hot meat dishes; ale or stout; mineral waters. |

No wine allowed in either case.

We think most housekeepers would fail to detect any reason why No. 1 should cost three or four times as much as No. 2. The breakfast is identical, since the small, items mentioned on the nurses' side are provided as a matter of course at the doctors' table. The doctors' lunch corresponds pretty closely to the nurses' supper; the doctors' supper to the nurses' tea; the dinner remains, and here it is hard to trace any serious difference. Poultry once a week accounts for a little extra cost, and the soup or fish item has to be reckoned. For 2d., or at most 3d., per head excellent soup can be served, and at a cost of from 4d. to 6d. per head a good choice of even the better kinds of fish. Thus even if fish or soup were a daily luxury they need not add more than about 2s. 6d. per head to the weekly average. But the actual difference is from 15s. to 20s.

It may be argued plausibly that men require more food than women. In ordinary households this is certainly the case, but experience has shown that hardworked nurses require fully a man's portion, and that no appreciable difference can be noted between their consumption of food and that of male members of the household who are served from the same table. This factor should, however, we think, be allowed a certain place in estimating expenditure. The fact is that the startling contrasts noticeable in hospital finance can nearly always be traced back to minute variations of circumstances and custom, and when several of these variations occur simultaneously the result, as in the present case, will appear out of all proportion to the inducing cause.

One such trifling difference between the doctors' and nurses' table is the privilege which the doctors exercise in some hospitals of inviting guests to dinner. This practice, affording a fertile opening for extra expenditure, is one difficult to limit if once traditionally established, and is altogether incalculable by the housekeeper. Although more may not be served for the chance comers, it is certain that less will remain over, and the heads of institutions where this privilege is conceded would do well to scrutinise the extent to which it is used, and form a definite conception of the extra cost incurred.

In minor respects it may be divined that the diet of the officers is managed with less attention to economy in details, and with less regard to discipline than that of the rest of the household. A very slight relaxation of the laws of economy tells instantly on the weekly average for provisions. Lamb instead of mutton, salmon instead of cod, sea-kale instead of cauliflower. Camembert cheese instead of American, such simple changes would suffice to double the cost of quite an ordinary repast, without the introduction of any really expensive articles of diet.

Again it is a truism that the heaviest additions to expenditure are commonly produced by mere careless handling of cheap necessaries. Milk and mineral waters, most laudable and inexpensive of drinks, will vanish to a truly alarming extent when no limit is placed on their supply, and odd meals at all hours, such as the medical officers of large institutions are apt to find indispensable, are another invitation to laxity.

Taking all things into account, some additional expense in providing this part of the household as distinct from the remainder will be found to be due to natural causes, and therefore inevitable. But the percentage on the total cost, as it works out in some of those institutions where it is ascertainable, is clearly unsatisfactory. Whether it is to the interests of either the hospital or the medical officers that a fancy price should be paid for their board in lieu of salary is a matter which gravely concerns the hospital finance of the future.

